# Dual Targeting of DNA Damage Response Proteins Implicated in Cancer Radioresistance

**DOI:** 10.3390/genes14122227

**Published:** 2023-12-17

**Authors:** Spyridon N. Vasilopoulos, Hüseyin Güner, Merve Uça Apaydın, Athanasia Pavlopoulou, Alexandros G. Georgakilas

**Affiliations:** 1 DNA Damage Laboratory, Physics Department, School of Applied Mathematical and Physical Sciences, National Technical University of Athens (NTUA), Zografou Campus, 15780 Athens, Greece; svasilopoulos@acg.edu; 2 Department of Science and Mathematics, Deree-The American College of Greece, 6 Gravias Street, 15342 Athens, Greece; 3 Izmir Biomedicine and Genome Center (IBG), 35340 Izmir, Turkey; huseyin.guner@ibg.edu.tr (H.G.); merve.uca@ibg.edu.tr (M.U.A.); 4 Izmir International Biomedicine and Genome Institute, Dokuz Eylül University, 35340 Izmir, Turkey; 5 Department of Molecular Biology and Genetics, Faculty of Life and Natural Science, Abdullah Gül University, 38080 Kayseri, Turkey

**Keywords:** radiation therapy, radiation resistance, DNA damage repair, computer-aided drug design, dual targeting, molecular dynamics

## Abstract

Ionizing radiation can induce different types of DNA lesions, leading to genomic instability and ultimately cell death. Radiation therapy or radiotherapy, a major modality in cancer treatment, harnesses the genotoxic potential of radiation to target and destroy cancer cells. Nevertheless, cancer cells have the capacity to develop resistance to radiation treatment (radioresistance), which poses a major obstacle in the effective management of cancer. It has been shown that administration of platinum-based drugs to cancer patients can increase tumor radiosensitivity, but despite this, it is associated with severe adverse effects. Several lines of evidence support that activation of the DNA damage response and repair machinery in the irradiated cancer cells enhances radioresistance and cellular survival through the efficient repair of DNA lesions. Therefore, targeting of key DNA damage repair factors would render cancer cells vulnerable to the irradiation effects, increase cancer cell killing, and reduce the risk of side effects on healthy tissue. Herein, we have employed a computer-aided drug design approach for generating ab initio a chemical compound with drug-like properties potentially targeting two proteins implicated in multiple DNA repair pathways. The findings of this study could be taken into consideration in clinical decision-making in terms of co-administering radiation with DNA damage repair factor-based drugs.

## 1. Introduction

Cancer represents one of the most formidable challenges in modern medicine and is the leading cause of death worldwide. According to the World Health Organization (WHO)/International Agency for Research on Cancer, there were approximately 10 million cancer-related deaths in 2020 globally, and a significant increase in cancer cases worldwide is expected over the next twenty years. The current cancer treatment modalities include surgery, chemotherapy, radiation therapy, hormonal therapy, immunotherapy, and targeted therapy [1].

Radiotherapy, which utilizes ionizing radiation (IR) to induce DNA damage and ultimately destroy cancer cells, is a common treatment for many types of solid cancers, including glioblastoma, lung, breast, rectal, prostate, colorectal, cervical, esophageal, and head and neck cancers [1,2]. Approximately 50% of cancer patients undergo radiotherapy at some stage of their treatment, usually combined with surgery and/or other therapeutic approaches [2,3]. Recent advancements in radiotherapy have markedly improved its therapeutic effect [4].

IR-induced DNA damage can be either direct or indirect. In the former type of damage, radiation causes intracellular DNA lesions such as abasic sites, single-strand breaks (SSBs), and double-strand breaks (DSBs) [5], which have a deleterious effect and may also lead to chromosomal rearrangements [6]. In the latter type, absorption of IR by water molecules in the cells results in the generation of reactive oxygen species (ROS) that cause cellular stress and eventually DNA damage [7,8]. 

Different types of DNA lesions are processed via specific pathways, where the cells sense DNA damage and respond accordingly [9]. DSBs are repaired mainly by homologous recombination (HR) and non-homologous end joining (NHEJ) [10]. SSB repair is considered to be a sub-pathway of base excision repair (BER), which is responsible for repairing normal and abnormal strand breaks [10]. It has been shown that IR is capable of inducing clusters of DNA lesions, including SSBs, DSBs, oxidized base lesions, as well as regular and oxidized abasic sites [11]. This complex DNA damage is crucial for the irradiated cells’ genomic instability and cell death. Furthermore, it poses a challenge to the cell’s DSB- and non-DSB-related repair mechanisms [12]. For example, BER has been shown to repair clustered lesions with a reduced efficiency as compared to the isolated lesions [13].

Cancer cells, like normal cells, respond to DNA damage caused by IR through DNA damage response and repair (DDR/R) signaling pathways. DDR/R is considered a critical determinant in radioresistance, in terms of tumor cell survival upon exposure to IR. It has been demonstrated that genes/proteins involved in the DDR/R pathways, apoptosis, hypoxia, and metabolism play an important role in tumor radioresistance [14,15,16]. 

Tumor cells’ radioresistance poses a major hurdle to the radiotherapy treatment outcome. Many patients undergoing radiotherapy develop resistance, which is associated with poor prognosis [17]. The administration of drugs to increase the sensitivity of cancer cells to IR (i.e., radiosensitization) constitutes a very promising approach to improving the efficacy of radiotherapy in cancer treatment. The combination of conventional chemotherapeutic agents (e.g., platinum-based drugs) with radiotherapy is a standard curative treatment for many cancer patients, but results in severe side effects in many cases [18]. Contemporary strategies focusing on targeting pathways that regulate response to radiation at the cellular level are currently under intensive investigation. Such strategies include inhibition of DNA repair, induction of cell death pathways, suppression of survival pathways, and p53 reactivation [19]. Targeting DDR-associated molecules renders cancer cells more sensitive to the genotoxic effects of IR, as they accumulate DSBs and other forms of non-DSB DNA damage. DDR inhibitors and their clinical applications have been reviewed extensively, and their combination with radiotherapy and immunotherapy shows promising results for cancer prognosis. Of note, in a clinical setting, a tumor is very rarely irradiated a second time. Thus, the development of radioresistance induced by radiotherapy is not a problem since no combination of radiotherapy and drugs increasing the sensitivity of cancer cells to radiation is considered. This contraindication is based on the intolerance of healthy peritumoral tissues to a second exposure to radiation. On the other hand, almost all glioblastoma patients do not respond significantly to radiation. For these reasons, drugs inhibiting DDR pathways are clinically relevant [17,20]. 

In our study, an in silico methodology was employed towards designing a novel drug-like molecule targeting two undruggable DDR/R-related proteins, that is, replication protein A2 (RPA2) and MutL homolog 1 (MLH1), which have been demonstrated to participate in DNA repair pathways and contribute to cancer development and progression. 

## 2. Materials and Methods

### 2.1. Structure-Based De Novo Dual Target Drug Design

#### 2.1.1. Target Proteins

The resolved tertiary structure of the target protein RPA2 was obtained from the Protein Data Bank (PDB), San Francisco, CA, USA [21]; PDB ID: 8A43, Chain B. MLH1 (NCBI RefSeq ID: NP_000240) has no resolved structure, and therefore, AlphaFold/ColabFold (https://github.com/sokrypton/ColabFold; accessed on 27 July 2023) [22] was used to predict its corresponding three-dimensional (3D) structure.

#### 2.1.2. Ligand Building

LigBuilder V3 [23] was employed for the design of drug-like ligands potentially binding to the RPA2 and MLH1 proteins. The ‘Cavity’ module of this program was used to detect ligandable binding pockets on the surface of the two proteins, and the ‘de novo’ mode was applied for the generation of novel chemical compounds potentially binding to the cavities of both proteins by using the default parameters. 

#### 2.1.3. Ligand Compound Naming 

ChemDraw (https://revvitysignals.com/products/research/chemdraw; accessed on 23 August 2023) and the SMILES format converter, Smi2Depict (https://cdb.ics.uci.edu/cgibin/Smi2DepictWeb.py; accessed on 23 August 2023), were used to generate the chemical structure of the ligand along with its chemical properties.

#### 2.1.4. Drug-Likeness, Bioavailability, and Toxicity Evaluation

The essential pharmacokinetic properties and drug-likeness of the generated ligand compound, that is, absorption, distribution, metabolism, excretion (ADME), as well as bioavailability, were further evaluated using SwissADME [24], which is an online tool that provides free access to a collection of robust predictive models for pharmacokinetics and drug-likeness.

The freely available tools *e*ToxPred [25] (based on a machine learning algorithm) and pkCSM [26] (based on key toxicity parameters) were employed to reliably evaluate the toxicity risk of the novel ligand.

### 2.2. Molecular Dynamics Simulations

Atomistic molecular dynamics (MD) simulations on protein–ligand complexes were conducted with specific focus on ligand binding to the RPA2 and MLH1 proteins separately. The selection of these protein–ligand complexes was guided by pharmacophore analysis. To carry out these simulations, GROMACS version 2021.4 (https://doi.org/10.5281/zenodo.5636567; accessed on 10 January 2022) was employed as our simulation engine. Standard and well-established MD analysis protocols were followed so as to ensure the comprehensive exploration of the entire molecular system. 

To clean and prepare the initial complexes for MD analysis, a Python (http://www.python.org; accessed on 10 September 2023) script was utilized that incorporates the dockprep routine provided by Chimera [27]. This script also facilitated the addition of missing residues with the assistance of the Dunbrack rotamer library. In order to prepare the ligand and generate the corresponding topology files compatible with our chosen forcefield, OPLS-AA/M, the LigParGen tool was employed.

In the initial stage of the MD analysis, the prepared ligand was merged with the cleaned protein structure and subsequently a water box was introduced by employing the SPCE water model. The topology files of both the protein and the ligand were incorporated into the system. 

The complex of 1.2 Å was placed away from the center of a dodecahedron-shaped water box and filled with the water molecules based on the initial model selected. To ensure the system’s neutrality, counterions of both sodium (Na) and chloride (Cl) were introduced at a physiological concentration of 0.15 M.

The MD simulations were initiated by conducting an energy minimization of the entire system using the steepest descent algorithm, involving a total of 50,000 steps. Following this, we proceeded with system equilibration in two stages. The first equilibration stage involved maintaining a constant volume (NVT ensemble) at a temperature of 300 K for 100 picoseconds (ps). Subsequently, we continued with a second equilibration step, this time at a constant pressure (NPT ensemble), also for 100 ps.

After the two equilibration phases, an extensive MD simulation was carried out, extending over a duration of 50 nanoseconds (ns). The simulation was conducted with a time step of 2 femtoseconds (fs) at a constant temperature of 300 K and atmospheric pressure of 1 bar. During this MD run, the energy values and coordinates were recorded every 10 picoseconds (ps). For pressure coupling, the Parrinello–Rahman method [28] was employed, and for temperature coupling, the V-rescale approach [29] was utilized.

### 2.3. Calculation of Molecular Interaction Free Energies

In this study, the molecular mechanics Poisson–Boltzmann surface area (MM-PBSA) method [30] was employed to determine the molecular interaction free energies (ΔG) within the ligand–protein complexes. The MM-PBSA approach was implemented utilizing the program *g_mmbsa*, which integrates functionalities from both Gromacs and APBS programs. This computational method allowed us to quantitatively evaluate the energetic aspects of ligand–protein interactions within our system, thereby providing valuable insights into the binding affinity and stability of these complexes.

In our calculations, we specified the solvent and solute dielectric constants as 80 and 4, respectively, to accurately account for the electrostatic and solvation effects in the system. Trajectories were sampled at regular intervals of 1000 picoseconds (ps) to perform the MM-PBSA calculations, enabling the construction of a comprehensive profile of the binding energy evolution throughout the simulation.

### 2.4. Molecular Visualization 

The molecular images were generated using the PyMOL Molecular Graphics System, version 2.5.4, Schrodinger LLC (https://newsite.schrodinger.com/pymol/; accessed on 20 March 2023). LigPlot2 was also used for the generation of two-dimensional (2D) diagrams of the protein–ligand interactions [31].

### 2.5. Virtual Screening of DDR/R proteins 

#### 2.5.1. Chemical Compound Selection and Structure Preparation

We prepared our target chemical space from the Comprehensive Natural Products Database (COCONUT) [32] and subsequently filtered the dataset to discover potential drug candidates. The three-dimensional structures of the selected compounds were generated using the RDKit release 2023.09.1 (http://www.rdkit.org/; accessed on 25 October 2023) Python library. Additionally, we incorporated partial charges and protonation states for the newly constructed ligands to enhance their structural accuracy.

#### 2.5.2. Pocket Identification and Analysis

To explore potential binding sites for the identified compounds, we utilized Fpocket [33], a powerful tool for investigating regions of interest within proteins that may interact with small chemicals.

#### 2.5.3. Docking Experiments

Selected pocket regions were subjected to a series of docking experiments on both Replication Protein A1 (RPA1) and MutL homolog 1 (MLH1). The docking simulations were executed using smina [34], a robust docking engine and a clone of Autodock Vina [35]. To streamline and automate the workflow, we implemented these experiments within a Nextflow [36] workflow developed in-house. This comprehensive approach allowed us to systematically explore the binding interactions between our selected compounds and the target proteins, providing valuable insights into potential dual drug candidates.

## 3. Results

### 3.1. In Silico Drug Design

In our study, the drug design program LigBuilder V3 [23] was employed in order to create a ligand compound targeting both RPA2 and MLH1. The ‘Cavity’ module implemented in this program allows users to discover binding sites in the target proteins, by taking into consideration the pharmacophoric features of the protein and calculating the druggability of the detected binding regions. In our study, the detected cavity with the highest druggability score was selected, and subsequently the ‘de novo’ option of LigBuilder V3 was chosen for creating new compounds that potentially bind to the detected binding sites of both RPA2 and MLH1 target proteins; this mode does not require a user-defined “seed” structure to be pre-placed into the binding site of the target protein, but instead an sp^3^ carbon with four hydrogen atoms is randomly inserted into the binding pocket, serving as the core for the progressive construction of a new molecule by applying local energy minimization at each stage. A genetic algorithm implemented into LigBuilder V3 is used for building ligands. In order to create a bigger compound that would fit into the active sites of the two proteins, organic fragments (i.e., building blocks) were first chosen from the default fragment library of LigBuilder V3 and joined to the seed structure with a synchronous growth operation. The new “seed structure pool” is created by the recombined fragments of the next generation of compounds, which are descended from the parent population. These fragments are chosen to function as “seeds” in the following cycles of ligand design because they are statistically more fit (i.e., privileged) than their parents. The general process is repeated until convergence, i.e., until all novel, ideal ligand molecules have been produced.

The generated ligand compounds are assessed automatically based on a number of factors, including (i) the lock–key model, which was used to evaluate the conformational complementarity of proteins and ligands; (ii) calculation of the ligand–protein binding affinity, based on the predicted ligand’s average binding affinity for each target; (iii) possession of particular physicochemical properties that would facilitate protein–ligand chemical specificity through the binding pocket; and (iv) synthesis accessibility of the newly-built ligand compound [37]. LigBuilder V3 incorporates pertinent functional modules, including filtering of toxic fragments and Lipinski’s rule of five (RO5) [38] (i.e., molecular weight, polar surface area, number of rotatable bonds, hydrogen bond donors/acceptors, and octanol–water partition coefficient), in order to further evaluate the drug-likeness of the ligand compound.

The best-scoring novel compound that targets both RPA2 and MLH1 is 2-(4-amino-3-(7-hydroxy-1H-benzo[d]imidazole-4-carbonyl)phenyl)acetic acid with the chemical formula C_16_H_13_N_3_O_4_ and molecular weight of 311.297 g/mol (Figure S1). This compound exhibits drug-like properties since it is non-toxic, it has no more than one violation of Lipinski’s RO5, and it is synthesizable (Figure S2). In particular, a favorable bioavailability score of 0.55 [39] and high gastrointestinal (GI) absorption (Figure S2A) were predicted for the ligand compound, highlighting the suitability of the ligand for oral administration. Also, a synthetic accessibility score of 2.49 (in a 1 (easy synthesis) to 10 (very difficult synthesis) rating scale [40]) (Figure S2A) suggests that the ligand can be synthesized relatively easy. A toxicity score of 0.55 was computed for the novel compound with *e*ToxPred, indicative of a non-toxic molecule, as the cut-off value for discriminating effectively non-toxic from toxic molecules is 0.58 [40]. In addition, based on the pkCSM output, the ligand is not predicted to cause hepatotoxicity, skin sensitization, or potential mutagenicity (i.e., AMES toxicity [41]). The estimated maximum tolerated dose 0.451 log(mg/kg/day) is also less than 0.477 log(mg/kg/day) [42], which is considered to be non-toxic to humans (Figure S2B).

### 3.2. DDR Protein–Ligand Interaction Sites

An MD simulation system containing the RPA2 and MLH1 target proteins in complex with the newly designed ligand was set up; several simulations of both the perturbed (protein–ligand) and unperturbed (protein without ligand bound to it) system were performed in order to assess the consistency of the simulation results [43].

**Figure 1:**
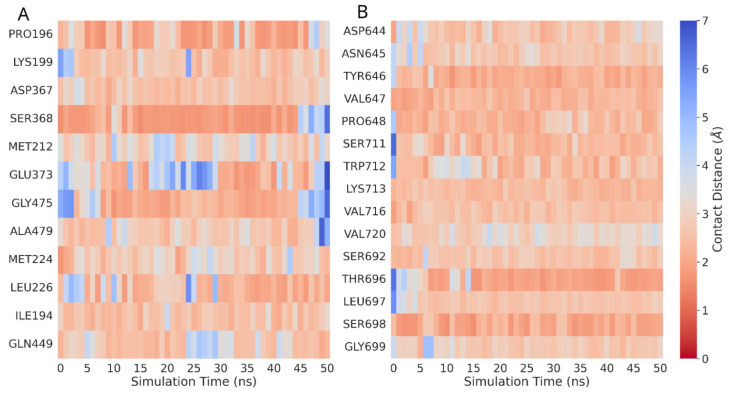
Close contact heatmap. Residue–ligand interactions by minimum distance close contacts heatmap of interacting residues in (**A**) RPA2 and (**B**) MLH1 with the ligand based on minimum distance per residue. Red: distant contact; blue: close contact.

Heatmaps were generated by using the distance matrix derived from spatial measurements obtained during MD trajectory analysis in order to visualize close contacts between residues in RPA2 and MLH1 and the designed ligand. The residues chosen to be displayed on the heatmap are those involved in interactions during the entire 50 ns simulation, identified by an average minimum distance to any ligand atom less than 3.5 Å. The principal interacting elements of the protein are depicted throughout the entire simulation, where blue and red hues indicate more or fewer close contacts (Figure 1).

**Figure 2:**
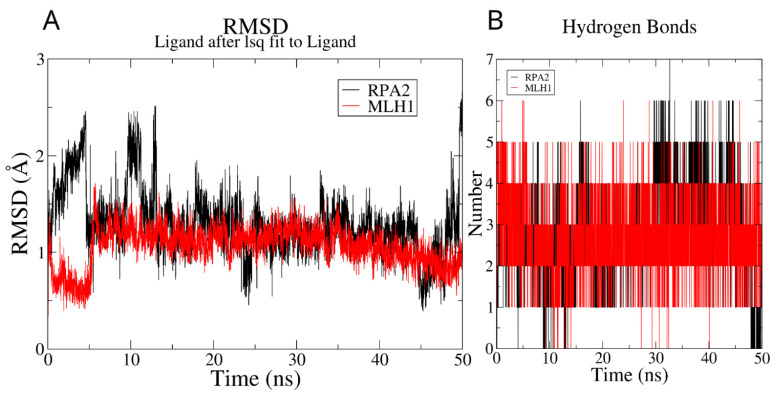
RMSD and hydrogen bond analysis on the MD trajectory: (**A**) ligands’ RMSD profiles, calculated using least squares fitting to their initial structures throughout the simulation period; (**B**) examination of hydrogen bond formation.

The RMSD analysis of the 50 ns MD simulation revealed that, around 6 ns into the simulation, the newly designed ligand consistently converged to stable conformations. This convergence signifies the establishment of stable molecular arrangements, demonstrating the overall stability of the ligands throughout the simulation (Figure 2A). The radius of gyration for the ligand molecule in two distinct complex formations, as illustrated in Figure S3, demonstrates a similar convergence pattern derived from RMSD analysis. The fluctuation in the number of hydrogen bonds formed during the simulations is illustrated in Figure 2B, revealing a range from 2 to 6 for MLH1 and 1 to 5 for RPA2. Throughout the majority of timepoints, MLH1 consistently maintained at least three hydrogen bond interactions, whereas RPA2 sustained two hydrogen bonds. This dynamic observation underscores the varying hydrogen bond profiles between the ligand and the two proteins, with MLH1 generally exhibiting a higher number of hydrogen bonds compared to RPA2 during the simulation course.

**Figure 3:**
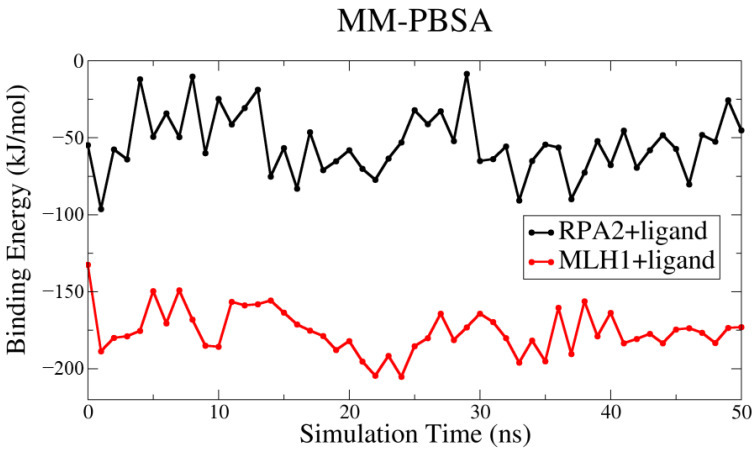
The binding energies of the protein–ligand complexes in kJ/mol were computed using the MM-PBSA methodology.

Figure 3 provides a comprehensive depiction of the dynamic behavior of the ligand interacting with two distinct proteins, RPA2 and MLH1. Despite the minor fluctuations observed, the overall trajectory of the binding energy unfolds in a consistent linear progression. This pattern across the entire spectrum of binding energies suggests a stable and sustained interaction, emphasizing the reliability and persistence of the ligand–protein interactions throughout the simulation. In light of the presented results in Table 1, the novel ligand, designed through delicate pharmacophore analysis, demonstrates a versatile binding profile. Despite a relatively weaker binding affinity with RPA2 (−54.04 kJ/mol), the ligand exhibits a notably stronger binding affinity with MLH1 (−175.48 kJ/mol). This versatility underscores its potential as a promising candidate for dual targeting, with favorable interactions observed for both protein targets. 

**Figure 4:**
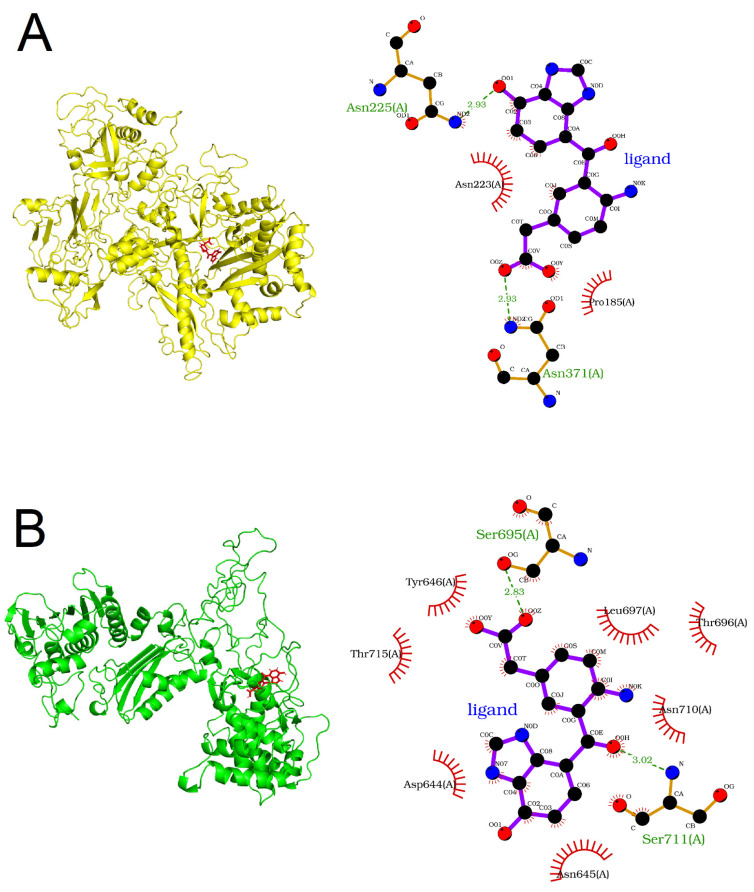
Protein–ligand binding poses for (**A**) RPA2 and (**B**) MLH1. Left: cartoon representation of target proteins and stick representation of the ligand. Right: 2D view of the ligand binding sites.

The protein–ligand complexes, illustrating the binding poses produced at the end of their individual MD simulations, are shown in Figure 4. The amino acids Asp185, Asn223, Asn225, and Asn371 in RPA2 were found to interact with the ligand (Figure 4A,B) based on LigPlot2. According to InterPro version 97.0 [44], an integrative database of functionally important protein domains and sites, the three asparagine residues are found to reside in Rpb2, the ‘lobe’ domain of the RNA polymerase (InterPro ID: IPR007642), into the concave surface of which DNA fits during transcription [45]. Moreover, the carboxy-terminal domain of Mlh1 (InterPro ID: IPR032189), part of the endonuclease active site [35], harbors the residues Asp644, Asn645, Tyr646, Ser695, Thr696, Leu697, Asn710, Ser711, and Thr715, which appear to interact with the ligand compound (Figure 4A,B). 

### 3.3. Natural Compound Analogs

Following a delicate examination of identified binding regions, taking into consideration their inhibitory role in DNA binding and the proximity of their location to respective biological domains, we determined Pocket 1 for MLH1 and Pocket 84 for RPA2 as our target search spaces. To precisely define the 3D grid coordinates of these selected binding pockets, we employed Pymol scripting. We filtered the COCONUT chemical library based on the RO5, resulting in a refined set of 43,131 natural products exhibiting diverse origins, sources, and chemical classifications. Subsequently, employing the Nextflow workflow for docking campaigns on both proteins, we acquired structure files and docking score log files for each natural compound, capturing their top nine conformers. Setting a minimum threshold of −9 kcal/mol, we identified lead candidates by evaluating the docking scores of the best conformers for each compound with both proteins. Post-application of the −9 kcal/mol threshold, a manual curation process was conducted. This process led to the identification of 11 common natural compounds, showcased in Table S1, which exhibit the potential to function as dual inhibitors. These compounds demonstrate promising inhibitory properties and are poised as strong candidates for further investigation and development.

## 4. Discussion

Computational and “omics”-based approaches have accelerated the discovery of biomarkers and potential therapeutic targets in many diseases, including cancers [46]. In a study by Toy and colleagues (2021), bioinformatic approaches were used to analyze “omics” data, resulting in the identification of 36 ‘radiogenes’, which are differentially expressed between radioresistant and radiosensitive cancer cells, and could be considered as potential targets for enhancing radiosensitization of cancer cells [3]. In fact, many research efforts focus on DNA repair inhibitors as an attractive strategy for enhancing the effectiveness of chemotherapy and radiotherapy. Several of those inhibitors have been introduced in clinical trials and others show promising results in the pre-clinical stage. It has been proposed that combining DDR inhibition with radiotherapy can improve cancer patient prognosis in clinical practice [20,47]. 

Targeting and modulating two different receptors with one single ligand (dual targeting) represents an attractive strategy for efficient cancer treatment, and is associated with reduced drug dosage and prevention of off-target drug–drug interactions [48,49,50]. Herein, we focused on the in silico investigation of dual targeting of the proteins RPA2 and MLH1, which are implicated in several cancer-relevant DDR pathways [47].

The *RPA2* gene encodes the RPA32 protein, which is a subunit of the Replication Protein A (RPA) trimeric complex (RPA70, RPA32, and RPA14), which plays an important role in DNA replication, DNA damage repair, and cell cycle regulation [51]. The RPA protein complex binds to SSBs with high affinity [52]. Regarding its role in DNA repair, RPA has been reported to interact with a variety of protein factors and participate in major DNA repair pathways, including nucleotide excision repair (NER), BER, MMR, and DSB repair via HR [53,54]. In response to DNA damage, the RPA2 subunit is hyperphosphorylated by the family of phosphatidylinositol 3-kinase-related kinases (PIKKs), thereby facilitating mitotic exit and DNA repair [55,56]. In NER, many studies have reported that RPA participates both in damage recognition and in the incision and gap-filling reactions [57,58]. In HR, it has been shown to interact with Rad52 and Rad51 [53,59] and it is suggested to be part of two core resection machineries, namely, BLM–DNA2–RPA–MRN and EXO1–BLM–RPA–MRN [60]. 

IR causes a variety of DSB and non-DSB DNA lesions, which are processed by the appropriate pathways, and tumor radioresistance greatly depends on the ability of cancer cells to repair the IR-induced DNA damage [16]. Because of its critical role in DNA repair, RPA is believed to be involved in the effectiveness of radiotherapy [16] and could act as a potential biomarker for the preliminary assessment of immunotherapy in brain tumors like glioblastoma [61,62]. 

Abasic sites, oxidized bases, and DSBs are common types of DNA lesions caused by IR, and RPA actively participates in the corresponding repair mechanisms, namely NER and BER for abasic sites and oxidized bases and HR for DSBs [58,63]. Therefore, we can easily assume that RPA is involved in tumor radioresistance by affecting the capacity of tumor cells to repair the various types of DNA damage caused by IR, such as DSBs and oxidative clustered DNA lesions [3,53]. RPA2 overexpression has been observed in many cancers, suggesting its potential role as a prognostic factor [64,65,66]. Di and colleagues (2014) demonstrated that RPA1 or RPA2 silencing increased the radiosensitivity of radioresistant esophageal cancer cells. In addition, it was shown that RPA1 and RPA2 silencing resulted in cell cycle arrest in G2/M phase, rendering cells more vulnerable to the effects of radiation, thereby contributing further to radiosensitivity [67]. 

Targeting RPA represents a potential therapeutic approach in cancer treatment as it as has been shown by a few recent studies. HAMNO ((1Z)-1-[(2-hydroxyanilino)methylidene]naphthalen-2-one), TDRL-505, and fumaropimaric acid (NSC15520) are small molecule inhibitors that have been demonstrated to target the RPA70 subunit of RPA in vitro [68,69,70,71]. HAMNO is the most studied, but it has shown controversial results regarding its effect on the radiosensitization of cancer cells. Pedersen et al. (2020) reported that RPA inhibition by HAMNO resulted in increased radiosensitivity of glioblastoma cancer stem-like cells [62]. A recent study by Dueva and colleagues (2023) reported no significant changes in the survival of HAMNO-treated irradiated lung carcinoma cells [72], whereas Feng et al. (2023) demonstrated that RPA inhibition by HAMNO increased the radiosensitivity of nasopharyngeal carcinoma cells, and this is a promising result because radiotherapy is the standard therapeutic approach for nasopharyngeal carcinoma [73]. Collectively, the aforementioned indicate that the RPA inhibition effects could be cell-type specific. 

The *MLH1* gene encodes the MLH1 protein, which is part of the DNA mismatch repair (MMR) pathway. MMR contributes to the maintenance of genome integrity by recognizing and repairing base mismatches that arise during DNA replication, recombination, and chemical or physical DNA damage [74]. The histidine kinase-like ATPase domain of MLH1 is also required for DNA end processing of DSBs through canonical NHEJ [75].

A relationship between MMR pathway defects and human cancers is well documented in the cancer predisposition Lynch syndrome/hereditary non-polyposis colorectal cancer (LS/HNPCC) [76]. Mutations in the *MLH1* and *MSH2* genes constitute the majority of mutations in HNPCC [77]. Recent studies also verify the relationship of MMR-related genes with sporadic cancers [78]. Epigenetic silencing of *MLH1* has been associated with increasing rates of mutation accumulation in cancers [79,80]. The epigenetic silencing of the *MLH1* gene via methylation of its promoter is a typical example of MMR deficiency in many tumors, leading to microsatellite instability (MSI) [81]. Although the role of MLH1-mediated MMR in DNA damage response to IR and radioresistance has been investigated, it is not yet very well characterized. In a study by Huang and coworkers (2022), *MLH1^+^* human colorectal cancer cells exhibited increased resistance to IR as compared to *MLH1^–^* cells [82]. The MMR protein MSH2, which is implicated in the processing of clustered DSBs and non-DSBs, as well as in apoptotic cell death elicited by IR [83], has been found to be upregulated in radioresistant cancer cells [3]. Currently, there are no MMR-specific inhibitors for cancer treatment [47]. 

Therapeutic agents that target DNA repair generally constitute a promising approach in cancer treatment [47,84]. Molecular targets in DNA repair that are currently being investigated in pre-clinical and clinical trials include DNA PKcs, ATM, ATR, PARP, CHK1, and WEE1 [84]. Inhibition of these targets has been shown to confer to the radiosensitivity of tumor cells [61]. 

In our study, we designed an easily synthesizable ligand compound, with favorable pharmacokinetic properties and without potential toxicity risk, which is capable of targeting both RPA2 and MLH1. This drug-like compound could potentially modulate the functions of the target proteins, since it appears to interact with amino acids important for their structure and activity, with high affinity (Figure 3 and Figure 4). This ligand is also predicted to have several structural analogues of natural compounds that show high binding potential (Table S1). These agents can be isolated from natural sources at relatively low cost, and are considered potential bioavailability enhancers [85,86].

A major advantage of dual targeting is that it can lead to synthetic lethality, a phenomenon in which, while the occurrence of a single genetic event is non-lethal, the co-occurrence of multiple genetic events results in cell death [87]. In the context of DNA repair, we could describe synthetic lethality as a phenomenon where the inhibition of two or more DNA repair pathways leads to increased cell death, while inhibition of either pathway alone does not. The synthetic lethal interaction between PARP inhibition and BRCA mutations offered a new therapeutic perspective for BRCA-mutant tumors, driving further research into synthetic lethality approaches in cancer treatment [84,88]. An example of a synthetic lethality approach, with the usage of two distinct DDR inhibitors, is presented in a recent study by Patterson-Fortin and coworkers (2022), wherein targeting of NHEJ and microhomology-mediated end-joining (MMEJ) pathways induced toxic DNA damage to *TP53*-deficient tumors [89]. Another example of such an approach is the combined inhibition of CHK1 and WEE1 that was found to be cytotoxic for head and neck squamous cell carcinoma cell lines [90]. As previously mentioned, targeting RPA showed controversial, cell type-specific results. Thus, the concurrent targeting of both RPA2 and MLH1 could increase the efficacy of radiotherapy in cancers in which RPA inhibition alone has shown poor results, assuming intolerable toxicity for organisms; the latter merits further investigation.

Apart from the increased target efficacy, our proposed dual targeting approach could reduce the likelihood of cancer cells developing resistance to radiotherapy. It is known that cells become dependent on alternative pathways to repair DNA if the primary DDR pathways are perturbed or defective [89]. Given that both RPA2 and MLH1 have been identified as multi-pathway proteins [47], targeting both key players simultaneously would affect various signaling pathways, possibly resulting in increased tumor-killing efficacy and radiosensitization of cancer cells at an IR dose lower than the standard one, minimizing in this way any toxic side effects. Furthermore, taking into account the complexity and genetic variability of cancers, this novel ligand might be effective across different types of cancers that are likely to exhibit variations in the functional status of their DDR pathways [91,92]. For example, a recent genomic data analysis of colorectal cancer patients revealed variations in the DDR-related mutations, and it is suggested that the DDR status could serve as a predictive marker [93]. 

These findings could be extrapolated in the field of personalized precision medicine, taking into consideration the genetic profiling of cancer patients, towards co-administering this drug-like ligand agent with radiotherapy and/or other types of cancer treatment. Recent studies show that cancer therapies like radiotherapy and other types of therapies that damage DNA remodel the tumor immune microenvironment, offering the possibility of applying immunotherapy to tumors that are resistant to this type of therapy [61]. IR therapy promotes the release of tumor neoantigens during cancer cell death increasing the immunogenicity of the tumor, and it also promotes the activation of cytotoxic T-cells and dendritic cells [94,95]. Especially for the MMR system that is targeted by our designed ligand, it has been found that the MMR and MSI status of the tumor can predict the patient’s response to immunotherapy in combination with the standard therapy. MSI leads to a high mutational load and production of neoantigens that trigger the host immune response and increase the density of tumor-infiltrating lymphocytes [81,96]. Currently, there are ongoing clinical trials to evaluate the effect of immune checkpoint blockade therapies (anti-PD-1/PD-L1) in many types of MMR-deficient tumors [96]. 

Exposure to IR used in radiotherapy causes DNA fragment leakage into the cytoplasm and the formation of micronuclei. The presence of cytosolic DNA and micronuclei has been found to activate the immune response via the cGAS-STING pathway that induces type I interferon production [97]. Combination of radiotherapy and immunotherapy (radioimmunotherapy) can be potentially considered as a promising multimodal clinical approach in cancer treatment, and the rationale for this combination is the immunogenic effect of radiotherapy [97,98]. Combining radiotherapy with DNA repair inhibitors and immunotherapy is reported to be a promising approach to improving the prognosis of cancer patients [20]. It is in this context that novel ligands, such as the one proposed by our group, can be considered as possible candidates for introduction to pre-clinical and clinical trials. 

## 5. Conclusions

In conclusion, our study extends previous research on DDR/R targeting in relation to resistance to radiation or other genotoxic agents. We suggest a novel promising approach towards increasing the efficacy of possible drugs towards tumor targeting. Within the scope of this study, we designed a drug-like ligand compound aiming at two multi-pathway DDR proteins. The ligand’s ability to manifest robust binding with distinct proteins highlights its potential applicability as a radiosensitizing agent for accelerating DNA damage in irradiated cancer cells, by potentially modulating the activity of the DDR proteins. The findings of this study could be a first step for further exploration of the novel ligand in drug development endeavors, as well as exploitation in clinical decision-making regarding the co-delivery of radiotherapy and DDR-targeting drugs in animals or patients assuming overall acceptable levels of adverse treatment effects.

## Figures and Tables

**Table 1:** MM-PBSA energy values.

Energies (kJ/mol)	RPA2 + Ligand	MLH + Ligand
Van der Waal	−80.69 ± 2.27	−185.46 ± 1.64
Electrostatic	−64.74 ± 4.01	−94.66 ± 2.31
Polar solvation	106.51 ± 3.30	122.22 ± 1.57
SASA	−15.13 ± 0.25	−17.58 ± 0.12
Binding	−54.04 ± 2.77	−175.48 ± 1.98

## Data Availability

Publicly available datasets were analyzed in this study.

## Supplementary Materials

The following supporting information can be downloaded at https://www.mdpi.com/article/10.3390/genes14122227/s1, Figure S1: Chemical structure of the generated drug-like ligand compound; Figure S2: Screenshot of the output of (A) SwissADME and (B) pkCSM; Figure S3: RMS fluctuation and radius of gyration measurement on the MD trajectory: (A) ligands’ RMS fluctuation profiles per residues for RPA2 and MLH1 and (B) radius of gyration plots of only two ligand structures; Table S1: Docking scores (kcal/mol) of selected natural compounds.
