# Supplementary figures and images for: Dual Targeting of DNA Damage Response Proteins Implicated in Cancer Radioresistance
Source: Genes (Basel). 2023 Dec 17;14(12):2227. doi: 10.3390/genes14122227 (PMC10742610; doi:10.3390/genes14122227)

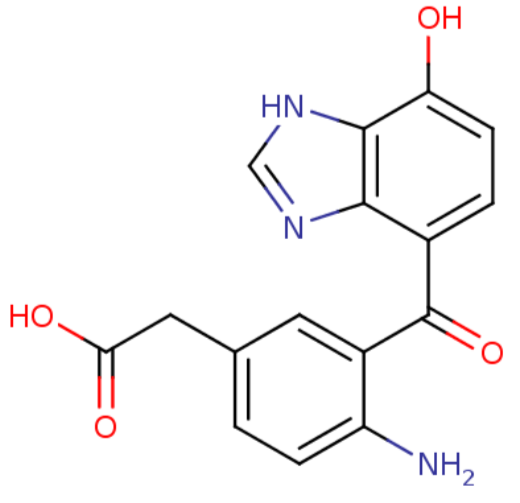

Supplement: Supplementary file 1 [file genes-14-02227-s001.zip › FigureS1.png]

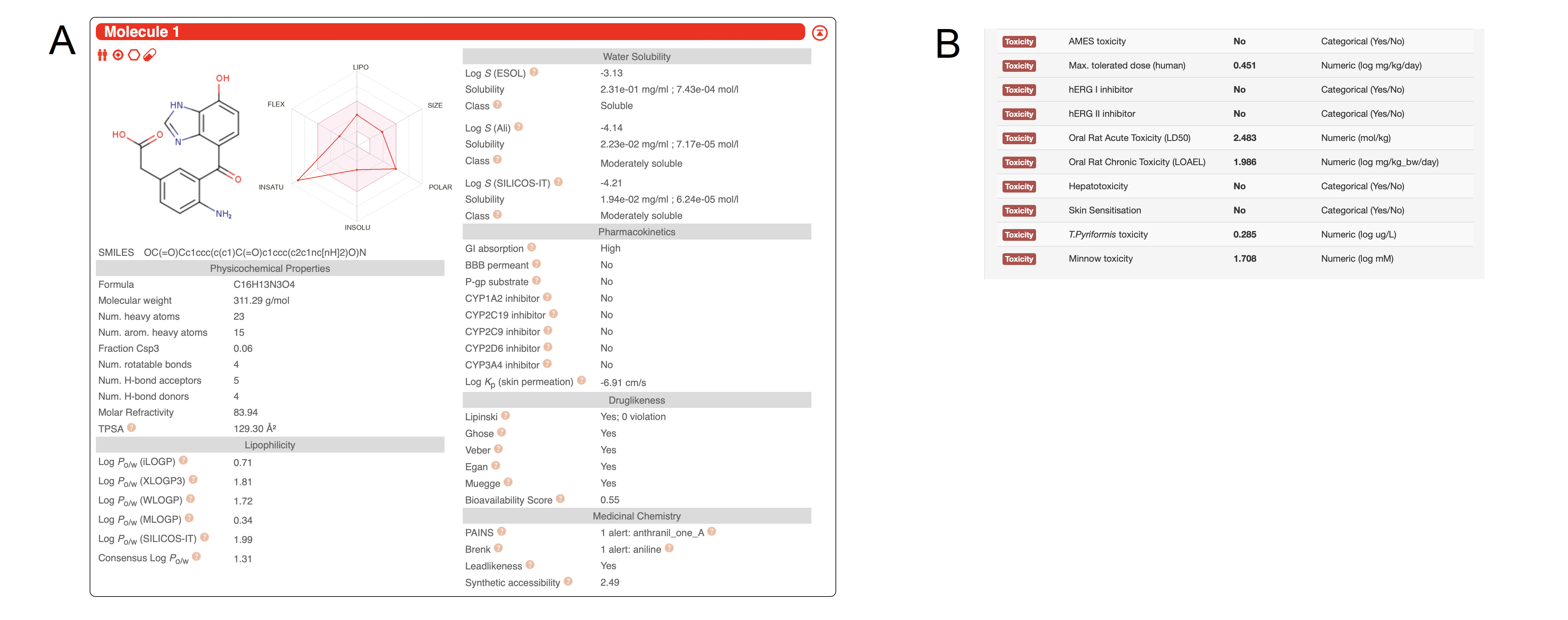

Supplement: Supplementary file 1 [file genes-14-02227-s001.zip › FigureS2.png]

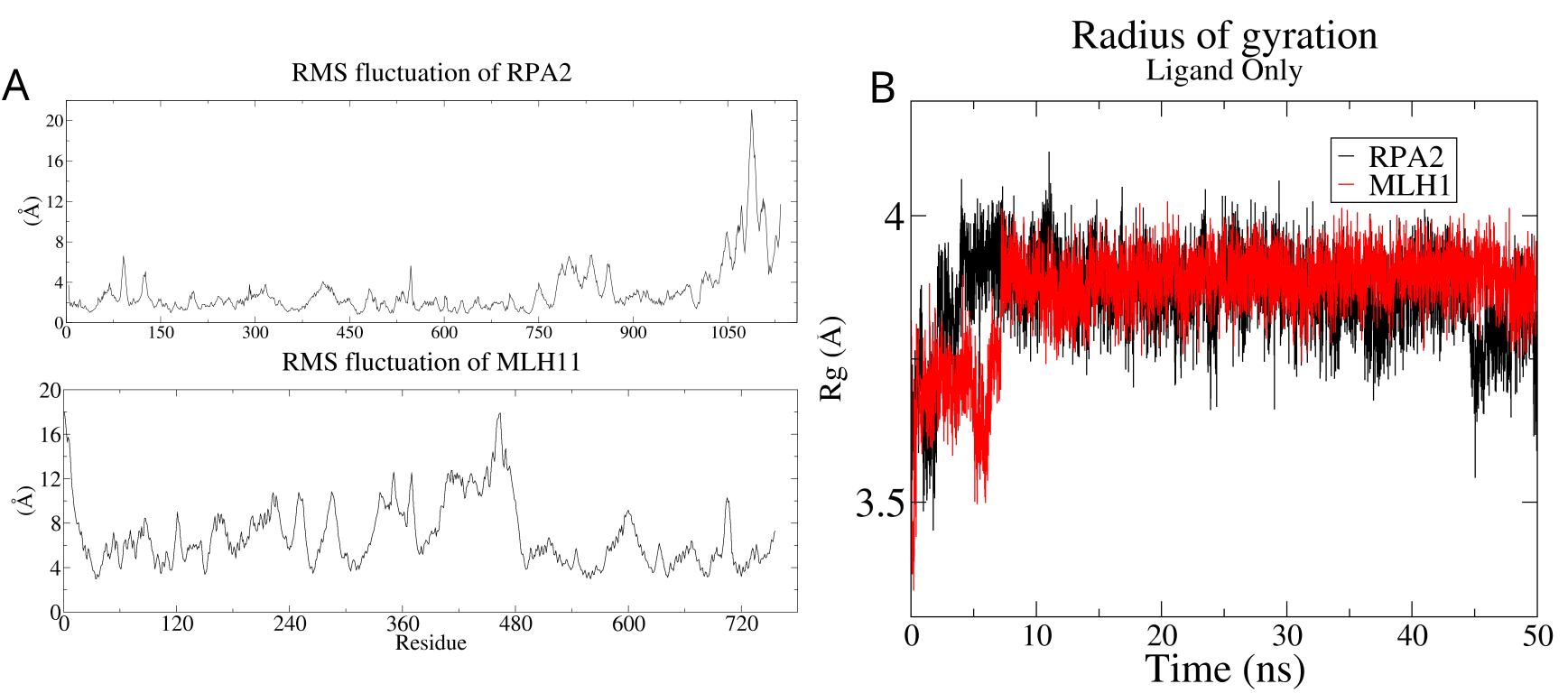

Supplement: Supplementary file 1 [file genes-14-02227-s001.zip › FigureS3.tif]

| COCONUT CID | MLH1 | RPA2  | 2D IMAGE                                                                             |
|-------------|------|-------|--------------------------------------------------------------------------------------|
| CNP0185875  | -9.1 | -10.1 | 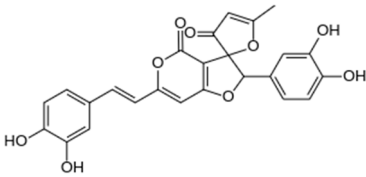   |
| CNP0094896  | -9.2 | -10.2 | 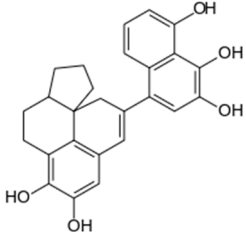   |
| CNP0090072  | -9.2 | -9.2  | 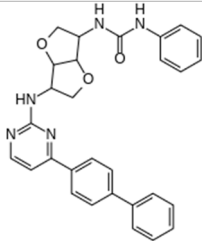  |
| CNP0220750  | -9.3 | -9.2  | 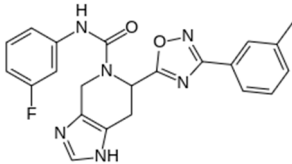 |
| CNP0255376  | -9.4 | -10   | 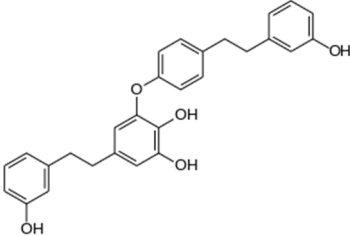 |
| CNP0318409  | -9.5 | -10.7 | 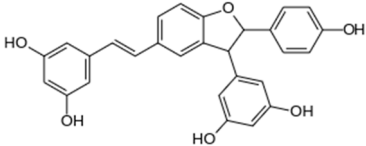 |
| CNP0161809  | -9.5 | -9.6  | 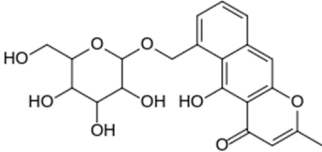 |

|            |       |       |                                                                                     |
|------------|-------|-------|-------------------------------------------------------------------------------------|
| CNP0197331 | -9.6  | -10.3 | 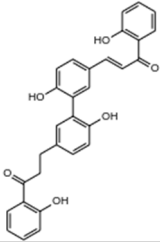  |
| CNP0086971 | -9.6  | -10.4 | 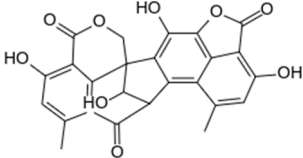  |
| CNP0214126 | -9.7  | -9.7  | 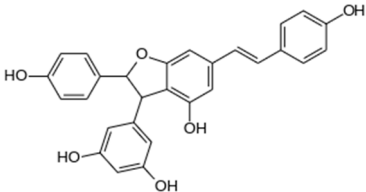  |
| CNP0150856 | -10.2 | -9.3  | 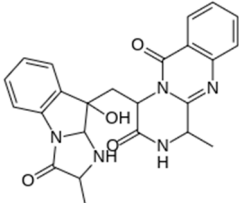 |

Supplement: Supplementary file 1 [file genes-14-02227-s001.zip › TableS1.pdf]
